# Molecular mechanisms and preclinical evidence of natural products in diabetic osteoporosis: a review

**DOI:** 10.3389/fendo.2026.1793925

**Published:** 2026-03-12

**Authors:** Tongyi Zhou, Yunfeng Yu, Shuo Yang, Fan Xiao, Rong Yu, Guomin Zhang

**Affiliations:** 1 College of Integrated Chinese and Western Medicine, Hunan University of Chinese Medicine, Changsha, China; 2 School of Traditional Chinese Medicine, Hunan University of Chinese Medicine, Changsha, China; 3 Hunan Key Laboratory of Traditional Chinese Medicine Prescription and Syndromes Translational Medicine, Changsha, China

**Keywords:** angiogenesis, diabetic osteoporosis, gut-bone axis, natural products, osteoimmunology, programmed cell death

## Abstract

Diabetic osteoporosis (DOP) is a prevalent and severe skeletal complication of diabetes, characterized by reduced bone mass, impaired bone microarchitecture, and elevated fracture risk. Its pathogenesis involves multiple interconnected mechanisms, including hyperglycemia-induced accumulation of advanced glycation end products (AGEs), oxidative stress, chronic inflammation, immune dysregulation, and programmed cell death of bone-related cells. Conventional therapies targeting single pathways often yield limited benefits, underscoring the need for alternative approaches. Traditional natural products and herbal formulations have long been used as complementary strategies for DOP prevention and treatment, exhibiting multifaceted protective effects. Experimental studies have demonstrated that bioactive compounds derived from these products can attenuate AGEs toxicity, reduce oxidative stress, modulate inflammation within the bone microenvironment, and regulate osteoblast and osteoclast apoptosis, autophagy, ferroptosis, and pyroptosis, thereby restoring the balance between bone formation and resorption. Some compounds also influence epigenetic regulation and cellular senescence and indirectly contribute to bone remodeling through modulation of gut microbiota, bone angiogenesis, and endocrine signaling. Stimulus-responsive delivery systems have been investigated to enhance bioavailability and bone-targeting efficiency. This review provides a comprehensive and updated overview of natural products and their active constituents in DOP management, highlighting underlying molecular mechanisms and potential therapeutic targets to support rational application and drug development.

## 1 Introduction

Diabetic osteoporosis (DOP) is a major skeletal complication of diabetes mellitus (DM) and is closely associated with increased fracture risk and adverse clinical outcomes (1, 2). With global population aging and the continuous rise in diabetes prevalence, DOP has become an increasingly important clinical concern (3–5). Epidemiological studies have demonstrated that individuals with diabetes exhibit a significantly higher overall fracture risk compared with non-diabetic populations, particularly at critical weight-bearing sites such as the hip (6–8). Notably, fracture-related mortality is also elevated in patients with diabetes (9, 10). Moreover, Clinical assessment of DOP is further complicated by the so-called “bone density paradox” (11), whereby some patients with diabetes present with normal or even increased bone mineral density (BMD) yet remain at substantially elevated fracture risk (12, 13). Consequently, conventional BMD-based evaluation tools or fracture risk calculators may underestimate the true skeletal fragility in this population (14–16). These observations suggest that DOP is not merely a disorder of reduced bone mass, but rather the result of complex remodeling of the bone microenvironment driven by chronic hyperglycemia. Multiple pathological processes are involved, including the accumulation of advanced glycation end products (AGEs), oxidative stress, chronic low-grade inflammation, dysregulated programmed cell death of bone cells, and systemic metabolic disturbances. Current clinical management strategies combine glycemic control with anti-osteoporotic therapies; however, overall efficacy remains suboptimal (17, 18). Moreover, certain glucose-lowering agents have been reported to adversely bone metabolism, while the antifracture efficacy of antiresorptive therapies in diabetic populations remains to be established by adequately powered prospective randomized controlled trials (19–22). Within this complex pathological context, single-target pharmacological interventions often fail to achieve optimal skeletal protection.

Natural products and traditional herbal formulations, characterized by multi-component, multi-target, and multi-pathway regulatory properties, have shown unique potential in the management of complex chronic diseases. Increasing preclinical evidence indicates that natural products can exert bone-protective effects in diabetic animal models or high-glucose-induced cellular systems by modulating metabolic toxicity, oxidative stress, inflammatory signaling, and bone remodeling-related pathways. However, mechanistic findings remain fragmented and are largely derived from experimental models, and their integrative regulatory networks and translational potential have not yet been fully evaluated. Accordingly, this review summarizes recent advances in the mechanisms by which natural products intervene in DOP, focusing on metabolic regulation, oxidative stress, inflammatory immunity, bone cell fate, and multi-organ interactions, and further discusses current limitations and future research directions.

## 2 Attenuation of aberrant AGEs accumulation

### 2.1 Aberrant AGEs deposition in DOP

Persistent hyperglycemia-induced metabolic toxicity is widely recognized as a primary initiating factor in DOP. Among these mechanisms, the abnormal accumulation of AGEs and activation of the receptor for AGEs (RAGE) signaling axis constitute a critical pathological hub linking chronic hyperglycemia to impaired bone quality (23). AGEs are generated through non-enzymatic reactions between reducing sugars and proteins or lipids, and their formation and deposition in the bone matrix are markedly increased under hyperglycemic and metabolically dysregulated conditions (24, 25). AGEs compromise bone mechanical properties by inducing abnormal collagen cross-linking, thereby increasing susceptibility to microcrack formation (26, 27). In addition, AGEs activate downstream signaling pathways, including Phosphatidylinositol 3-kinase/protein kinase B (PI3K/Akt), Janus kinase/signal transducer and activator of transcription (JAK/STAT), mitogen-activated protein kinase (MAPK), and nuclear factor kappa-B (NF-κB), which promote oxidative stress and chronic inflammatory responses, suppress osteoblast function, and induce apoptosis, ultimately contributing to the low bone turnover phenotype characteristic of DOP. Both experimental and clinical evidence supports the pivotal role of AGEs in DOP. Animal studies have demonstrated that elevated AGEs levels in bone tissue correlate with reduced mechanical strength, whereas lowering glycation burden can partially restore bone strength (28). *In vitro* studies further indicate that glycated bone matrix is more prone to microcrack propagation (29). Clinically, increased AGEs-related markers have been detected in bone tissue and serum of patients with DM, showing a positive association with fracture risk but only a weak correlation with BMD (30, 31), thereby providing mechanistic insight into the “bone density paradox.” Based on these findings, inhibiting AGEs formation, enhancing their clearance, or blocking the AGEs–RAGE signaling axis has emerged as an important therapeutic strategy for DOP.

### 2.2 Natural products targeting abnormal AGE accumulation

Certain natural products reduce AGEs burden under hyperglycemic experimental models primarily by improving systemic glucose metabolism or directly inhibiting enzymatic processes involved in AGEs formation. For instance, PPCP-1, an arabinogalactan isolated from *Phellodendron chinense* Schneid., suppresses α-glucosidase activity and improves glucose tolerance, thereby reducing AGEs accumulation in streptozotocin (STZ)-induced diabetic rats. This effect is accompanied by downregulation of RAGE expression in bone tissue and attenuation of hyperglycemia-associated bone loss (32). These findings underscore that restoring glucose metabolic homeostasis and suppressing excessive AGE formation constitute a fundamental mechanism by which natural products exert skeletal protective effects against DOP.

Beyond limiting AGEs generation, multiple bioactive compounds directly interfere with AGEs–RAGE interactions or downstream pro-inflammatory and pro-apoptotic signaling cascades, thereby preserving osteoblast function under hyperglycemic conditions. Morroniside, derived from *Cornus officinalis*, enhances detoxification of the AGEs precursor methylglyoxal by activating glyoxalase 1 (Glo1) (33) and suppresses the AGEs/RAGE/p38 MAPK/NF-κB axis (34). Concurrently, it activates Wnt/β-catenin signaling, promoting osteogenic differentiation and maintaining osteoblast viability in high-glucose environments. Timosaponin AIII from *Anemarrhena asphodeloides* has been shown to form stable hydrogen-bonding and hydrophobic interactions with RAGE, thereby inhibiting RAGE/MAPK activation. This intervention reduces AGEs-induced expression of inflammatory cytokines such as Interleukin-1 beta (IL-1β), Interleukin-6 (IL-6), and Tumor Necrosis Factor-alpha (TNF-α), limits osteoblast apoptosis, and reverses diabetes-associated mineralization defects in zebrafish models (35). In addition, extracts of *Melastoma dodecandrum* and its active constituent isovitexin protect osteoblasts from AGEs-induced injury by enhancing antioxidant defenses, improving cell viability, upregulating osteogenic markers, and restraining excessive RAGE expression (36).

Compared with single-compound interventions, botanical extracts and traditional herbal formulas often exert multi-target synergistic effects, simultaneously modulating AGEs–RAGE signaling while integrating improvements in oxidative stress, inflammation, and mineral homeostasis. Aqueous extracts of *Mori Folium* suppress oxidative stress and inflammatory responses through regulation of the AGEs/RAGE/NADPH oxidase 4 (Nox4)/NF-κB axis, while maintaining calcium homeostasis via the Parathyroid Hormone (PTH)/Vitamin D Receptor (VDR)/calcium-binding protein pathway, thereby collectively improving trabecular architecture, material properties, and bone strength in diabetic rats (37). Extracts of *Epimedium brevicornum* reverse trabecular deterioration in DOP models by modulating key components of the AGE–RAGE pathway, suppressing inflammatory factor expression, and inhibiting osteoblast apoptosis (38). Compound formulations such as Osteoking promote osteogenesis and suppress osteoclastogenesis in db/db mice through activation of the AGEs/Insulin-like Growth Factor-1(IGF-1)/β-catenin/osteoprotegerin (OPG) axis, restoring skeletal balance (39). Qing’e Pill (QEP) improves bone mineral density and microstructural integrity in DOP rats by modulating AGE/RAGE signaling, exerting concomitant hypoglycemic and anti-inflammatory effects, and promoting osteogenesis and intraosseous angiogenesis (40). Similarly, SiJunZi Decoction (SJZD) enhances bone quality and mechanical performance by maintaining redox homeostasis, downregulating skeletal AGEs/RAGE/NF-κB signaling, and activating Wnt/β-catenin–mediated osteogenic pathways in db/db mice (41). Notably, accumulating evidence indicates that diverse natural products possess the capacity to reduce AGEs burden and mitigate their downstream skeletal effects (**Table 1**).

**Table 1:** Natural products attenuating AGEs accumulation in DOP.

Natural product	Source/Components	Model	Mechanisms	References
PPCP-1	*Phellodendron chinense* Schneid.	*In vivo*: STZ-induced rats	Inhibiting α-glucosidase activity;improving glucose tolerance and reducing AGEs accumulation	(32)
Morroniside	*Cornus officinalis* Siebold & Zucc.	*In vivo*: High-fat diet (HFD) and STZ-induced SD rats; *In vitro*: High glucose (HG)-induced BMSCs; AGEs-induced primary osteoblasts	Activating glyoxalase 1 (Glo1); inhibiting the AGEs/RAGE/p38 MAPK/NF-κB signaling axis and activating Wnt/β-catenin	(33, 34)
Timosaponin AIII	*Anemarrhena asphodeloides* Bunge	*In vivo*: Alloxan-induced DOP zebrafish model; *In vitro*: AGEs-induced primary rat osteoblasts	Inhibiting RAGE/MAPK signaling and downregulating pro-inflammatory cytokines expression including IL-1β, IL-6, and TNF-α	(35)
Isovitexin	*Melastoma dodecandrum* Lour.	*In vitro*: AGEs-induced osteoblasts	Enhancing antioxidant enzyme activity; upregulating osteogenic markers, and suppressing RAGE expression.	(36)
*Morus alba* leaf aqueous extract	*Morus alba* L.	*In vivo*: HG+HFD and STZ-induced Wistar rats	Modulating the AGEs/RAGE/Nox4/NF-κB and PTH/VDR/calcium-binding protein (CaBP) pathway; inhibiting oxidative stress and inflammation	(37)
*Epimedium brevicornum* extract (EPE)	*Epimedium brevicornum* Maxim.	*In vivo*: HG+HFD and STZ-induced rats	Regulating AGE-RAGE pathway and reducing inflammatory factor expression.	(38)
Osteoking(OK)	Pericarpium Citri Reticulatae, *Carthamus tinctorius* L., *Panax notoginseng* (Burkill) F.H.Chen, *Eucommia ulmoides* Oliv., *Panax ginseng* C.A.Mey., *Astragalus mongholicus* Bunge, Carapax Trionycis, *Rubus obcordatus* Franch., and *Datura metel* L. (Flos)	*In vivo*: db/db mice; *In vitro*: HG+AGEs treated MC3T3-E1 cells	Activating AGEs/IGF-1/β-catenin/OPG pathway.	(39)
Qing’e Pill (QEP)	*Eucommia ulmoides* Oliv., *Psoralea corylifolia* L., *Juglans regia* L., *Allium sativum* L.	*In vivo*: STZ-induced SD rats	Modulating AGE/RAGE pathway, Promoting osteogenesis and intraosseous angiogenesis	(40)
SiJunZi Decoction (SJZD)	*Panax ginseng* C.A. Mey., *Poria cocos* (Schw.) Wolf, *Atractylodes macrocephala* Koidz., and *Glycyrrhiza uralensis* Fisch. ex DC.	*In vivo*: HFD+STZ-induced mice	Downregulating AGEs/RAGE/NF-κB expression; activating the Wnt/β-catenin pathway	(41)

## 3 Suppression of oxidative stress

### 3.1 Oxidative stress in DOP

Oxidative stress is considered another key mechanism by which diabetic metabolic disturbances impair bone homeostasis. Persistent hyperglycemia promotes excessive reactive oxygen species (ROS) production through multiple metabolic pathways, leading to disruption of cellular redox balance (42, 43). Excessive ROS not only directly induce oxidative damage to DNA, lipids, and proteins but also function as aberrant signaling molecules that interfere with bone metabolic regulatory networks. This results in suppressed osteogenic differentiation, increased apoptosis of osteoblasts and osteocytes, and enhanced osteoclastogenesis, ultimately disrupting bone remodeling balance (44). Therefore, restoring redox homeostasis and strengthening endogenous antioxidant defense systems are regarded as important therapeutic approaches to mitigate DOP-related skeletal damage.

### 3.2 Natural products alleviating oxidative stress in DOP

Nuclear factor erythroid 2–related factor 2 (Nrf2) serves as a master transcriptional regulator of cellular antioxidant defense, orchestrating the expression of detoxifying and antioxidant enzymes such as heme oxygenase-1 (HO-1) and glutathione peroxidase 4 (GPX4). Accumulating evidence indicates that numerous natural products attenuate hyperglycemia-induced oxidative injury and improve bone metabolism by strengthening Nrf2-dependent protective responses. For instance, arctiin (45) and aqueous extracts of *Eucommia ulmoides* (46) significantly attenuate oxidative stress, promote osteogenic differentiation, and improve bone quality in diabetic models by enhancing Nrf2/HO-1 signaling. Resveratrol facilitates Nrf2 nuclear translocation through the Akt/Glycogen synthase kinase 3β (GSK3β) and FYN kinase (FYN) signaling axis and concurrently counteracts rosiglitazone-induced bone loss, highlighting its dual actions on metabolic regulation and skeletal protection (47, 48). Glabridin enhances Akt signaling and downstream phosphorylation events, lowers intracellular ROS levels, restores antioxidant enzyme activity, and consequently mitigates bone loss in diabetic models (49). Icariin preserves mitochondrial homeostasis by scavenging excessive ROS and subsequently supports osteogenesis via activation of cilia-associated signaling pathways (50). Consistently, compound formulations such as Jiangu Decoction exert antioxidant and osteoprotective effects through modulation of the Kelch-like ECH-associated protein 1 (Keap1)/Nrf2/HO-1 axis, as demonstrated in both cellular and animal models (51).

In addition to Nrf2-centered mechanisms, members of the sirtuin (SIRT) family—particularly SIRT1 and SIRT3—act as metabolic and stress sensors that play pivotal roles in maintaining mitochondrial integrity and redox balance. Natural products targeting SIRT signaling enhance the metabolic adaptability of bone cells under hyperglycemic conditions. Curcumin improves mitochondrial function and suppresses oxidative stress in osteoblasts through activation of the SIRT3/Forkhead box O3a(FoxO3a) pathway, an effect that is markedly attenuated upon SIRT3 silencing (52). Similarly, the osteoprotective effects of Qianggu Formula have been linked to activation of the SIRT1/Nrf2/HO-1 cascade and enhanced Nrf2 nuclear translocation (53).

Notably, certain natural products exert antioxidant effects through noncanonical pathways. Extracts of elderberry activate the Cyclic guanosine monophosphate (cGMP)/protein kinase G2 (PKG2) signaling pathway, reshaping the expression profile of redox-related genes and alleviating hyperglycemia-induced oxidative stress and bone loss (54). Moreover, emerging delivery strategies have further expanded the therapeutic potential of natural products. ROS-responsive nanocarriers loaded with proanthocyanidins enable site-specific drug release within high-ROS bone microenvironments, suppress NADPH oxidase 2 (NOX2)-mediated ROS generation, and promote angiogenesis–osteogenesis coupling, thereby substantially enhancing therapeutic precision and efficacy (55). These technological advances offer new strategies for improving the delivery efficiency and therapeutic utilization of natural products within the bone microenvironment; however, their long-term safety and clinical applicability remain to be further rigorous evaluated.

Collectively, by engaging multiple antioxidant pathways and reinforcing endogenous defense systems, natural products not only alleviate oxidative injury induced by hyperglycemia but also establish a permissive microenvironment that constrains downstream inflammatory responses, cell death, and remodeling imbalance in DOP (**Table 2**).

**Table 2:** Natural products inhibiting oxidative stress in DOP.

Natural product	Source/Components	Model	Mechanisms	References
Arctiin	*Arctium lappa* L.	*In vivo*: db/db mice; *In vitro*: HG-induced MC3T3-E1 cells	Activating Nrf2/HO-1 pathway	(45)
*Eucommia ulmoides* aqueous extracts	*Eucommia ulmoides* Oliv.	*In vivo*: HG+HFD and STZ-induced C57BL/6 mice	Activating Nrf2/HO-1 pathway	(46)
Resveratrol	*Vitis vinifera* L.; *Polygonum cuspidatum* Siebold & Zucc.	*In vivo*: HFD+STZ induced ovariectomized (OVX) SD rats; *In vitro*: HG-induced MC3T3-E1 cells	Promoting Nrf2 nuclear translocation via the Akt/GSK3β/FYN axis and reversing rosiglitazone-induced bone loss	(47, 48)
Glabridin	*Glycyrrhiza glabra* L.	*In vivo*: STZ-induced Wistar rats; *In vitro*: 2-deoxy-D-ribose treated MC3T3-E1 cells	Stimulating Akt phosphorylation; reducing ROS accumulation; and restoring antioxidant enzyme activity.	(49)
Icariin	*Epimedium* spp.	*In vivo*: STZ-induced SD rats; *In vitro*: HG-treated osteoblasts	Scavenging ROS; maintaining mitochondrial homeostasis and activating cilia-related signaling pathway	(50)
Jiangu Decoction (JGD)	*Rehmannia glutinosa* Libosch., *Epimedium brevicornu* Maxim., *Drynaria fortunei* (Kunze) J.Sm., *Cistanche deserticola* Y.C. Ma, *Astragalus mongholicus* Bunge, and *Angelica sinensis* (Oliv.) Diels.	*In vivo*: HFD and STZ-induced C57BL/6 mice;*In vitro*: HG-treated MC3T3-E1 cells	Regulating Keap1/Nrf2/HO-1 axis	(51)
Curcumin	*Curcuma longa* L.	*In vivo*: HG+HFD and STZ-induced SD rats; *In vitro*: HG-treated MC3T3-E1 cells	Activating SIRT3/FoxO3a axis and improving mitochondrial function.	(52)
Qianggu Concentrate	*Eucommia ulmoides* Oliv., *Astragalus membranaceus* (Fisch.) Bunge, *Cornus officinalis* Sieb. et Zucc., *Rehmannia glutinosa* (Gaertn.) DC., *Lycium barbarum* L., *Achyranthes bidentata* Blume, *Epimedium brevicornu* Maxim., *Cibotium barometz* (L.) J.Sm., *Psoralea corylifolia* L., *Dipsacus asperoides* C.Y.Cheng et T.M.Ai, *Ligusticum chuanxiong* Hort., and *Cistanche deserticola* Y.C.Ma.	*In vivo*: HG+HFD and STZ-induced SD rats; *In vitro*: HG-treated BMSCs	Activating SIRT1/Nrf2/HO-1 pathway and promoting Nrf2 nuclear translocation.	(53)
*Sambucus williamsii Hance* extracts	*Sambucus williamsii* Hance	*In vivo*: STZ-induced rats; *In vitro*: HG-treated MC3T3-E1 cells	Activating cGMP/PKG2 signaling pathway.	(54)
Proanthocyanidin(loaded nano-system)	*Pinus pinaster* Aiton; *Vitis vinifera* L.	*In vivo*: db/db mice; *In vitro*: HG and palmitate-induced MC3T3-E1 cells	Exhibiting ROS-responsive release, downregulating NOX2, and promoting angiogenesis-osteogenesis coupling.	(55)

## 4 Modulation of the bone inflammatory and immune microenvironment

### 4.1 Chronic inflammation and immune dysregulation in DOP

The development and progression of DOP are driven not only by direct metabolic insults to bone cells but also by a persistent state of systemic and local low-grade inflammation. Chronic hyperglycemia and metabolic stress-derived signals aberrantly activate immune responses within bone tissue, resulting in sustained activation of pro-inflammatory pathways including the NLRP3 inflammasome and NF-κB signaling (56). This maladaptive inflammatory microenvironment suppresses osteogenesis and induces inflammatory forms of programmed cell death, while simultaneously promoting osteoclast differentiation through upregulation of RANKL and multiple pro-inflammatory cytokines, thereby exacerbating bone remodeling imbalance (57, 58). Accordingly, restoration of the dysregulated immune and inflammatory microenvironment within bone has emerged as a critical strategy for interrupting the vicious cycle of bone destruction in DOP.

### 4.2 Natural products targeting the inflammatory and immune microenvironment of bone

The NLRP3 inflammasome represents a central molecular hub linking metabolic danger signals to inflammatory amplification. Its aberrant activation promotes caspase-1-mediated maturation of IL-1β and amplification of inflammatory cascades, thereby exacerbating inflammatory bone damage. Multiple studies have demonstrated that natural products can suppress aberrant inflammasome activation by interfering with upstream regulatory axes. Rosmarinic acid (RA), for instance, attenuates (Forkhead box O1) FOXO1/Thioredoxin-interacting protein (TXNIP)-mediated NLRP3 inflammasome activation, thereby preserving osteoblast function and improving bone mass and microarchitecture under high-glucose conditions (59). In parallel, RA directly suppresses the release of inflammatory mediators in RANKL-induced osteoclasts, leading to reduced bone resorptive activity (60). In addition, emerging evidence suggests that corylin, a bioactive isoflavone from *Cullen corylifolium*, indirectly inhibits excessive NLRP3 activation by targeting the DNA damage response protein Recombination activating gene 1 (RAG1) and reshaping related signaling networks, providing a novel molecular entry point for inflammasome-oriented intervention (61).

Beyond inflammasome signaling, sustained activation of canonical pro-inflammatory pathways also plays a pivotal role in driving bone homeostasis imbalance. A broad spectrum of natural products attenuates inflammatory signaling and restores the bone microenvironment through coordinated inhibition of NF-κB, MAPK, and related pathways. For example, bergapten protects trabecular architecture and suppresses osteoclastogenesis in diabetic osteoporosis models by inhibiting RANKL–RANK signaling and downstreaming activation of PI3K/Akt, JNK/MAPK, and NF-κB pathways (62). Sanhuang Jiangtang Tablet (SHJTT) interferes with osteoclast differentiation by suppressing the AKT–GSK3β–(TGF-β receptor type II)NFATc1 axis (63), while the herb pair *Rehmanniae Radix–Corni Fructus* (RR–CF) promotes osteogenesis via regulation of PI3K–Akt, TNF, and MAPK signaling pathways (64). Studies on velvet antler extract (VAE) suggest that its protective effects against DOP may be associated with modulation of Tumor Necrosis Factor (TNF)/PI3K–Akt signaling (65). Jingui Shenqi Pill has also been shown to ameliorate DOP by reducing the expression of inflammatory mediators such as IL-1β, IL-6, AGEs, and NF-κB in MC3T3-E1 cells (66).

Notably, the bone-protective effects of certain natural products reflect a coordinated interplay between anti-inflammatory actions and the promotion of bone formation. Mangiferin counteracts high-glucose–induced osteoblast dysfunction by activating PI3K/Akt signaling and inhibiting FOXO1-mediated transcriptional repression of SRY-Box Transcription Factor 9 (SOX9) (67). Combined treatment with Zuo Gui Wan and the active vitamin D analog ED-71 further enhances osteogenesis in db/db mice through synergistic activation of the PI3K/Akt pathway (68). Berberine improves bone microarchitecture and antagonizes pioglitazone-induced skeletal side effects by activating AMP-activated protein kinase (AMPK), upregulating osteogenic markers such as Runt-related transcription factor 2 (Runx2) and OPG, and concurrently dampening inflammatory signaling (69, 70). Osteoking not only promotes osteogenic differentiation in diabetic models but also significantly reduces circulating pro-inflammatory cytokines (IL-6, IL-17A, Interferon gamma[IFN-γ], TNF-α, and IL-1β) while increasing anti-inflammatory IL-10 levels, an effect closely linked to modulation of the PI3K/Akt/GSK-3β pathway (71). Gegen Qinlian Decoction exerts bone-protective effects by inhibiting the (Insulin-like growth factor-binding protein 3IGFBP3)/MAPK/NFATc1 axis, thereby suppressing osteoclast-related gene expression (72). Beyond inflammation suppression, certain natural products actively engage tissue repair and immune resolution pathways (73). Curcumin enhances the expression and activation of the Transforming Growth Factor beta 1/TGF-β receptor type I/TGF-β receptor type II/Mothers against decapentaplegic homolog 2/3 (TGF-β1/TβRI/TβRII/Smad2/3) signaling axis in DOP models, an effect strongly associated with improved bone microstructure and biomechanical properties (74). Bushen–Jianpi–Huoxue decoction synergistically activates Wnt/β-catenin–mediated osteogenic signaling while inhibiting NF-κB–driven inflammation (75). *Polygonatum sibiricum* polysaccharide (PSP) modulates arachidonic acid metabolism, peroxisome proliferator-activated receptor (PPAR) signaling, and amino acid metabolism while downregulating pro-inflammatory cytokines (TNF-α, IL-1β) in diabetic zebrafish models (76). Network pharmacology analyses further support the multi-target regulatory potential of natural products; for instance, active constituents of *Citri Reticulatae Pericarpium* have been predicted to alleviate DOP by targeting core inflammatory and oxidative stress–related nodes such as AKT1, MAPK1, and NF-κB (77).

In addition, emerging evidence highlights the capacity of certain natural products to modulate immune cell phenotypes and promote active resolution of inflammation. Chemically modified curcumin (CMC2.24) restores macrophage homeostasis in diabetic rats with comorbid periodontitis, reduces matrix metalloproteinase-9 and pro-inflammatory cytokine levels, and markedly upregulates resolvin D1 (RvD1), suggesting a pro-resolving mechanism that limits inflammation-driven bone loss (78). Rehmannia glutinosa polysaccharide not only ameliorates hyperglycemia and osteoporosis in diabetic mice but also rebalances splenic immune cell populations and enhances histone deacetylase 6 (HDAC6) expression in regulatory T cells, indicating an immunomodulatory contribution to its bone-protective effects (79). Indeed, accumulating evidence indicates that a wide range of natural products can exert distinct advantages in the prevention and treatment of DOP by targeting immune–inflammatory networks (**Table 3**).

**Table 3:** Natural products remodeling the inflammatory and immune microenvironment in DOP.

Natural product	Source/Components	Model	Mechanisms	References
Rosmarinic Acid (RA)	*Salvia rosmarinus* Spenn.	*In vivo*: HFD and STZ-treated mice; *In vitro*: HG-treated osteoclasts	Blocking NLRP3 inflammasome activation via FOXO1/TXNIP; protecting osteoblasts and inhibiting inflammatory factors	(59, 60)
Corylin	*Cullen corylifolium* (L.) Medik.	*In vivo*: STZ-treated mice; *In vitro*: LPS-treated BMSCs and BMMs	Targeting DNA damage response protein RAG1 to indirectly inhibit excessive NLRP3 inflammasome activation	(61)
Bergapten	*Citrus aurantium* L.; *Citrus bergamia* Risso	*In vivo*: HFD and STZ-treated C57/B6 and OPG(−/−) mice	Inhibiting RANKL–RANK signaling and blocking downstream activation of PI3K/Akt, JNK/MAPK, and NF-κB pathways	(62)
Sanhuang Jiangtang Tablet (SHJTT)	*Dioscorea polystachya* Turcz., *Glycyrrhiza uralensis* Fisch., *Pueraria montana* var. *lobata* (Willd.) Sanjappa & Pradeep, *Rehmannia glutinosa* (Gaertn.) DC., *Rheum palmatum* L., and *Astragalus mongholicus* Bunge.	*In vivo*: db/db mice	Suppressing AKT/GSK3β/NFATc1 signaling axis	(63)
*Rehmanniae Radix-Corni Fructus* Herb Pair (RR-CF)	*Rehmannia glutinosa* Libosch.; *Cornus officinalis* Siebold & Zucc.	*In vivo*: STZ-treated SD rats; *In vitro*: HG-treated MC3T3-E1 cells	Regulating multiple pathways including PI3K-Akt, TNF, and MAPK	(64)
Velvet Antler Extract(VAE)	*Cervus nippon* Temminck or *Cervus elaphus* Linnaeus	*In vivo*: HG+HFD and STZ-treated mice; *In vitro*: HG-treated MC3T3-E1 cells	Modulating TNF- and NF-κB–associated inflammatory signaling	(65)
Jingui Shenqi Pill	*Rehmannia glutinosa* Libosch., *Dioscorea oppositifolia* L., *Cornus officinalis* Siebold & Zucc., *Alisma plantago-aquatica* L., *Wolfiporia cocos* (F.A. Wolf) Ryvarden & Gilb., *Paeonia suffruticosa* Andrews, *Cinnamomum cassia* (L.) J. Presl, and *Aconitum carmichaelii* Debeaux.	*In vivo*: HFD-treated MKR mice; *In vitro*: HG-treated MC3T3-E1 cells	Reducing IL-1β and IL-6 expression, and modulating AGE/NF-κB signaling	(66)
Mangiferin	*Mangifera indica* L.	*In vitro*: HG-treated MC3T3-E1 cells	Activating PI3K/Akt and inhibiting FOXO1 to relieve suppression of SOX9	(67)
Zuogui Pill	*Rehmannia glutinosa* Libosch., *Lycium barbarum* L., *Dioscorea opposita* Thunb., *Cyathula officinalis* Kuan, *Cornus officinalis* Sieb. et Zucc., *Cervus elaphus* Linnaeus, *Cuscuta chinensis* Lam., and *Chinemys reevesii*.	*In vivo*: db/db mice; *In vitro*: HG-treated MC3T3-E1 cells	Synergizing with ED-71 to activate the PI3K/Akt pathway and enhance osteogenesis	(68)
Berberine	*Coptis chinensis* Franch.	*In vivo*: HFD and STZ-treated Wistar rats	Activating AMPK, upregulating Runx2 and OPG, and antagonizing pioglitazone’s adverse bone effects	(69, 70)
Osteoking (OK)	*Pericarpium Citri reticulatae*, *Carthamus tinctorius* L., *Panax notoginseng*, *Eucommia ulmoides* Oliv., *Panax ginseng*, *Astragalus mongholicus* Bunge, *Carapax trionycis*, *Rubus obcordatus*, and *Flos Daturae*	*In vivo*: db/db mice; *In vitro*: HG-treated MC3T3-E1 cells	Reducing pro-inflammatory cytokines (IL-6, IL-17A, IFN-γ, TNF-α, and IL-1β); increasing anti-inflammatory IL-10 levels; modulating PI3K/Akt/GSK-3β pathway	(71)
Gegen Qinlian Decoction (GQD)	*Pueraria montana* var. *lobata* (Willd.) Sanjappa & Pradeep, *Scutellaria baicalensis* Georgi, *Coptis chinensis* Franch., and *Glycyrrhiza uralensis* Fisch.	*In vivo*: db/db mice	Inhibiting the IGFBP3/MAPK/NFATc1 signaling axis and downregulating osteoclast-related gene expression	(72)
Curcumin	*Curcuma longa* L.	*In vivo*: HG+HFD and STZ-treated SD rats	Upregulating TGF-β1/TβRI/TβRII and promoting Smad2/3 phosphorylation	(73, 74)
Bushen-Jianpi-Huoxue Decoction (BJHD)	*Rehmannia glutinosa* Libosch., *Eucommia ulmoides* Oliv., *Astragalus membranaceus* (Fisch.) Bge., *Lycium barbarum* L., *Cornus cervi* (colla), *Salvia miltiorrhiza* Bge., *Anemarrhena asphodeloides* Bunge, and *Cyathula officinalis* Kuan	*In vivo*: HG-HFD and STZ-treated Wistar rats	Activating Wnt/β-catenin–mediated osteogenic signaling, inhibiting NF-κB–driven inflammation	(75)
*Polygonatum sibiricum Polysaccharide* (PSP)	*Polygonatum sibiricum* Redouté	*In vivo*: STZ-treated zebrafish	Regulating arachidonic acid metabolism, PPAR signaling, and amino acid metabolism; downregulating TNF-α and IL-1β	(76)
Chemically Modified Curcumin CMC2.24	A curcumin derivative	*In vivo*: STZ-treated SD rats *In vitro*: LPS-induced macrophage cells	Restoring macrophage homeostasis, reducing MMP-9 and pro-inflammatory cytokines, upregulating Resolvin D1 (RvD1)	(78)
Rehmannia glutinosa Libosch. Polysaccharide (RGP)	*Rehmannia glutinosa* Libosch.	*In vivo*: STZ-treated mice	Improving splenic immune cell balance and upregulating HDAC6 in regulatory T cells	(79)

## 5 Regulation of programmed cell death in skeletal cells

### 5.1 Programmed cell death of skeletal cells in DOP

During the progression of DOP, persistent hyperglycemia and its associated toxic microenvironment—characterized by excessive accumulation of AGEs and ROS—directly compromise the viability of key effector cells such as osteoblasts and osteocytes. This process constitutes the fundamental cellular basis for bone mass reduction, microarchitectural deterioration, and compromised biomechanical properties observed in DOP. Emerging evidence indicates that bone cell injury in DOP is not mediated by a single death modality but instead involves a complex and interconnected “cell death network” encompassing multiple forms of programmed cell death, such as ferroptosis, pyroptosis, apoptosis, and autophagy (80, 81). These pathways are interconnected and are often triggered by upstream factors such as oxidative stress (82).

### 5.2 Natural products regulating skeletal cell death in DOP

#### 5.2.1 Inhibition of ferroptosis

Ferroptosis is a form of programmed cell death characterized by iron-dependent abnormal accumulation of lipid peroxides (18). In DOP, hyperglycemic conditions suppress the System Xc^-^/glutathione (GSH)/GPX4 antioxidant axis, thereby impairing the capacity of osteoblasts to detoxify lipid peroxidation and predisposing them to ferroptotic death (83). A variety of natural products have been shown to restore GPX4 expression and reinforce antioxidant defenses primarily through activating Nrf2 nuclear translocation. Forsythiaside A (84) and poliumoside (85) both enhance Nrf2 activation and upregulate GPX4, effectively suppressing lipid peroxide accumulation. Asperosaponin VI exerts anti-ferroptotic effects via epigenetic regulation by inhibiting DNA methyltransferases 1 (DNMT1) and DNMT3a, thereby reversing hypermethylation of the Gpx4 promoter under diabetic conditions and restoring GPX4 protein expression (86). Additionally, proanthocyanidins (PACs) significantly improve trabecular bone microarchitecture, reduce bone marrow adiposity, and alleviate oxidative stress in DOP model mice by activating the SIRT6/Nrf2/GPX4 signaling axis (87). Notably, advanced nano-delivery strategies have further enhanced therapeutic efficacy; for instance, curcumin loaded onto a tetrahedral framework nucleic acid–based delivery system more efficiently activates the Nrf2/GPX4 pathway in bone marrow mesenchymal stem cells, synergistically inhibiting ferroptosis while promoting osteogenic differentiation (88).

#### 5.2.2 Attenuation of pyroptosis

Beyond inflammatory amplification, aberrant NLRP3 inflammasome activation also mediates caspase-1-dependent pyroptosis. Under hyperglycemic and oxidative stress conditions characteristic of DOP, this pathway promotes gasdermin D (GSDMD) cleavage, leading to osteocyte dysfunction and aggravated bone remodeling imbalance. Tanshinone IIA (Tan IIA) has been shown to attenuate osteoblast pyroptosis and bone loss in DOP models by blocking the inositol-requiring enzyme 1α (IRE1α)/TXNIP/NLRP3 signaling axis through inhibition of IRE1α, a key mediator of endoplasmic reticulum stress (89). Similarly, Extracts of the *Anemarrhenae Rhizoma/Phellodendri Chinensis Cortex* herb pair activate Nrf2 signaling while downregulating its negative regulator Keap1, enhancing global antioxidant capacity and indirectly suppressing aberrant NLRP3 inflammasome activation, which alleviates osteoblast pyroptotic injury in vertebral bone tissue (90).

#### 5.2.3 Modulation of apoptosis and autophagy

Hyperglycemic stress can trigger classical mitochondrial apoptosis in bone cells, whereas autophagy plays a context-dependent dual role in skeletal homeostasis. Basal autophagy facilitates clearance of damaged organelles and supports cell survival, while excessive or impaired autophagic flux may accelerate cell death. Maintaining a dynamic balance between apoptosis and autophagy is therefore critical for preserving bone cell function.

Puerarin mitigates high-glucose-induced inflammatory responses and apoptosis in osteoblasts by inhibiting the expression and activity of histone deacetylases 1 and 3 (HDAC1/HDAC3) (91). Timosaponin BII promotes protective autophagy by suppressing mammalian target of rapamycin (mTOR) phosphorylation and downstream NF-κB signaling, thereby alleviating mitochondrial oxidative stress and apoptosis induced by hyperglycemia (92). In contrast, Ziyin Bushen Formula inhibits pathologically excessive autophagy by reducing intracellular ROS levels and suppressing aberrant extracellular signal-regulated kinase (ERK) phosphorylation, indicating a bone-protective mechanism mediated through restraining detrimental autophagic overactivation (93). Furthermore, Xianling Gubao Capsule (XLGB) reverses high-glucose-induced suppression of osteoblast proliferation, migration, invasion, and osteogenic differentiation by activating the PI3K/Akt signaling pathway, facilitating cell cycle progression and inhibiting mitochondrial apoptosis (94).

**Figure 1:**
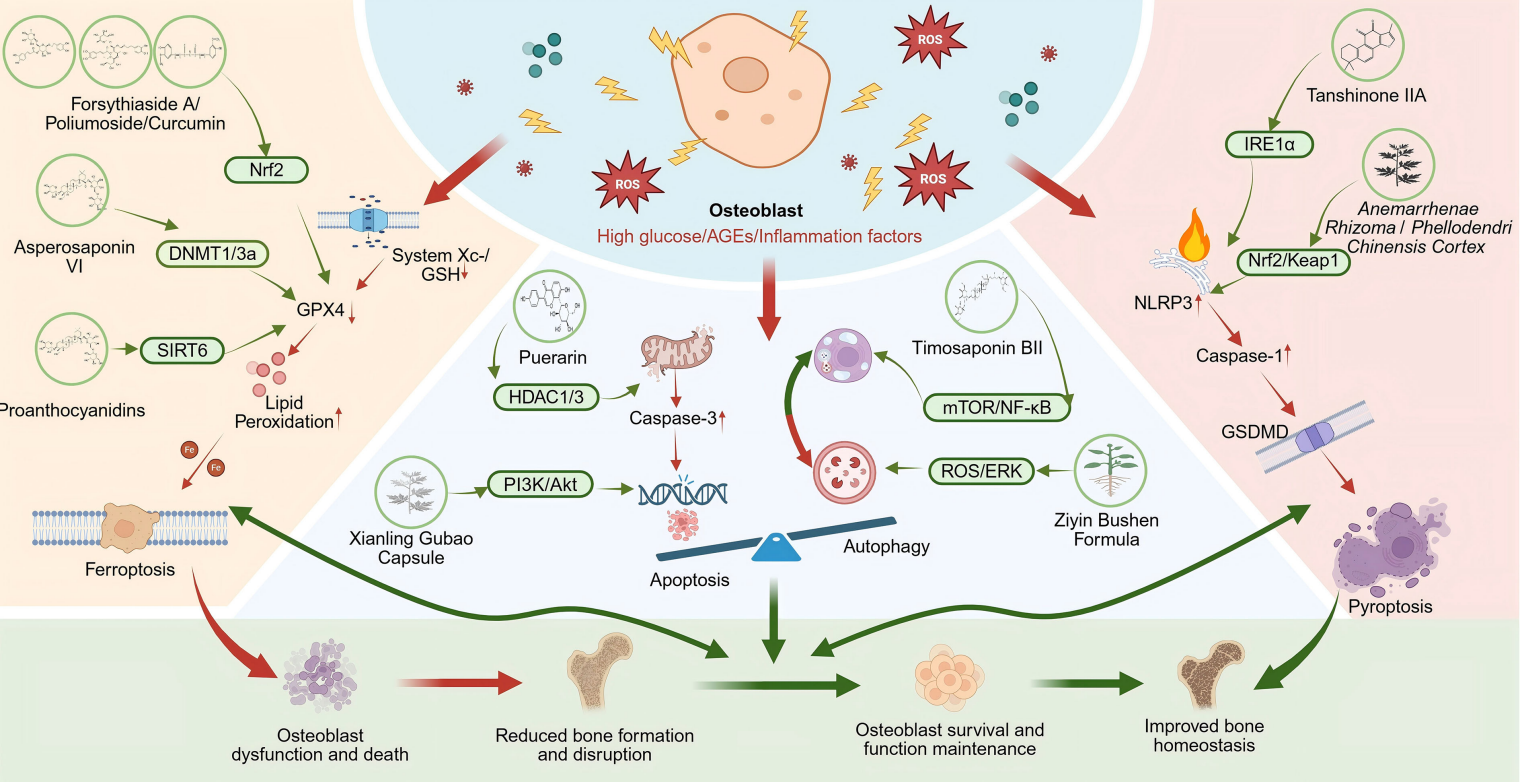
Molecular mechanisms of osteoblast programmed cell death and protective modulation by natural products in DOP. Red arrows indicate pathological activation; green arrows indicate protective regulation by natural products. Figure created with BioRender.com.

Consequently, selective modulation of specific cell death pathways has emerged as a key cellular mechanism through which natural products exert bone-protective effects in DOP. Representative compounds and their mechanisms of action are summarized in **Table 4** and illustrated in **Figure 1**.

**Table 4:** Natural products regulating bone cell programmed death in DOP.

Subtype/Natural product	Source/Components	Model	Mechanisms	References
Inhibition of ferroptosis				
Forsythiaside A	*Forsythia suspensa* (Thunb.) Vahl	*In vivo*: HG+HFD and STZ-induced SD rats; *In vitro*: HG-treated BMSCs	Promoting Nrf2 nuclear translocation, upregulating GPX4 expression, and inhibiting lipid peroxide accumulation.	(84)
Poliumoside	*Rehmannia glutinosa* (Gaertn.) Libosch.	*In vivo*: HG+HFD and STZ-induced murines; *In vitro*: HGHF-treated BMSCs	Activating the Nrf2/GPX4 pathway to suppress ferroptosis.	(85)
Asperosaponin VI	*Dipsacus asperoides* C.Y. Cheng et T.M. Ai	*In vivo*: HFD and STZ-treated C57BL/6J mice; *In vitro*: HG+palmitic acid-treated primary osteoblasts and osteoclasts	Inhibiting DNMT1/3a, reversing Gpx4 promoter hypermethylation, and restoring GPX4 expression.	(86)
Proanthocyanidins (PAC)	*Vitis vinifera* L.;*Pinus massoniana* Lamb.	*In vivo*: HG+HFD and STZ-induced mice; *In vitro*: HG-treated MC3T3-E1 cells	Activating the SIRT6/Nrf2/GPX4 axis, improving trabecular structure, and reducing bone marrow fat	(87)
Curcumin(loaded nano-delivery system)	*Curcuma longa* L.	*In vivo*: HFD and STZ-induced C57BL/6J mice; *In vitro*: AGEs-treated BMSCs	Efficiently activating the Nrf2/GPX4 pathway in BMSCs, inhibiting ferroptosis and promoting osteogenesis	(88)
Attenuation of pyroptosis				
Tanshinone IIA (Tan IIA)	*Salvia miltiorrhiza* Bunge	*In vivo*: HFD and STZ-induced C57BL/6J mice; *In vitro*: HG-treated MC3T3-E1 cells	Blocking the IRE1α/TXNIP/NLRP3 axis to mitigate osteoblast pyroptosis	(89)
*Anemarrhenae Rhizoma/Phellodendri Chinensis Cortex* extract	*Anemarrhena asphodeloides* Bunge/*Phellodendron chinense* Schneid.	*In vivo*: STZ-induced Wistar rats	Activating the Nrf2/Keap1 pathway to indirectly inhibit NLRP3 and alleviate pyroptosis	(90)
Modulation of apoptosis and autophagy				
Puerarin	*Pueraria lobata* (Willd.) Ohwi	*In vivo*: STZ-induced SD rats	Inhibiting HDAC1/3 to alleviate inflammation and apoptosis	(91)
Timosaponin BII	*Anemarrhena asphodeloides* Bunge	*In vivo*: GK rats *In vitro*: HG-induced osteoblast cells	Suppressing mTOR/NF-κB signaling, activating protective autophagy, and alleviating apoptosis	(92)
Ziyin Bushen Formula	*Os draconis*, *Ostrea rivularis* Gould, *Taxillus chinensis* (DC.) Danser, *Dipsacus asperoides* C.Y.Cheng & T.M.Ai, *Epimedium brevicornu* Maxim., *Trichosanthes kirilowii* Maxim., and *Coptis chinensis* Franch.	*In vivo*: HG+HFD and STZ-induced C57BL/6J mice; *In vitro*: HG-treated MC3T3-E1 cells	Reducing ROS and suppressing excessive ERK phosphorylation to inhibit detrimental autophagic overactivation	(93)
Xianling Gubao Capsule	*Epimedium brevicornu* Maxim., *Dipsacus asperoides* C.Y.Cheng & T.M.Ai, *Psoralea corylifolia* L., *Anemarrhena asphodeloides* Bunge, *Salvia miltiorrhiza* Bunge, *Rehmannia glutinosa* (Gaertn.) DC.	*In vitro*: HG-treated MG63 osteoblast-like cells	Activating PI3K/Akt, promoting cell cycle progression, and inhibiting mitochondrial apoptosis	(94)

## 6 Restoration of bone homeostasis

### 6.1 Bone homeostatic imbalance in DOP

The hallmark pathological feature of DOP is a profound imbalance between bone formation and bone resorption, characterized by impaired osteoblast function and abnormally enhanced osteoclast activity. Together, these alterations lead to a state of “low-turnover” bone loss (95, 96). Persistent hyperglycemia, excessive accumulation of AGEs, and a chronic inflammatory microenvironment suppress key osteogenic signaling pathways, including Wnt/β-catenin and bone morphogenetic protein (BMP) pathways, while aberrantly activating osteoclastogenic signaling cascades such as the RANKL/RANK axis (97). Moreover, diabetic metabolic disturbances drive a differentiation bias of bone marrow mesenchymal stem cells (BMSCs) toward adipogenic rather than osteogenic lineages, further exacerbating insufficient bone formation.

### 6.2 Natural products regulating bone homeostasis

#### 6.2.1 Promotion of osteoblast differentiation and bone formation

The Wnt/β-catenin signaling pathway is a central determinant of osteogenic lineage commitment in mesenchymal stem cells. In DOP models, icariin significantly improves bone mineral density, upregulates RUNX2 expression and the OPG/RANKL ratio in bone tissue, and promotes osteogenic differentiation of BMSCs by enhancing the expression of the transcription factor GLI Family Zinc Finger 1 (GLI1) (98). Aqueous extracts of *Marantodes pumilum* leaves upregulate skeletal Wnt3a and β-catenin while downregulating the inhibitory kinase GSK-3β, thereby ameliorating diabetes-related bone damage (99). Epigenetic regulation also plays an important role in osteogenic modulation. Catalpol (100, 101) and the classical formula Liuwei Dihuang Pill (LWDH) (102–104) promote osteoblast differentiation and improve bone microarchitecture and mechanical properties by inhibiting the histone demethylase Lysine demethylase 7A (KDM7A), thus relieving epigenetic suppression of the Wnt/β-catenin pathway.

The BMP/Smad signaling axis represents another essential osteogenic pathway. Total flavonoids extracted from *Rhizoma Drynariae* (RDF) markedly upregulate the expression of BMP2, Smad4/5, and RUNX2 in bone tissue of DOP rats, thereby activating BMP2/Smad signaling and promoting bone formation (105). In addition, multiple active components of *Rehmanniae Radix*—including verbascoside, and echinacoside—synergistically stimulate BMP signaling and the IGF-1/PI3K/mTOR pathway, collectively enhancing osteogenesis (106).

Diabetic bone disease is frequently accompanied by excessive bone marrow adiposity, reflecting a shift in BMSC differentiation from osteogenic to adipogenic lineages. Correcting this fate imbalance is therefore considered a key therapeutic direction in DOP. Baicalin promotes osteogenic differentiation while inhibiting adipogenesis of BMSCs through activation of the p38-MAPK pathway (107). Likewise, anthocyanin-rich extracts from black rice dose-dependently suppress bone marrow adipogenesis, elevate RUNX2 expression, and increase the OPG/RANKL ratio in bone tissue, thereby improving bone metabolic balance (108).

In addition, several natural products exert bone-protective effects by coordinately regulating osteogenesis- and osteoclastogenesis-related gene expression. The *Anemarrhenae Rhizoma/Phellodendri Chinensis Cortex* (AR/PCC) herb pair and its seven major blood-entering components (including alkaloids and flavonoid glycosides) improve bone microarchitecture in diabetic zebrafish and rat models by upregulating osteogenic genes (RUNX2b, OPG) while downregulating osteoclast-related genes (RANML) (109). Compounds such as quercetin (110), cinnamaldehyde (111), D-pinitol (112), and Epimedium polysaccharides (113, 114) have also demonstrated potential in improving bone density, microstructure, or osteogenic capacity in various diabetic bone disease models.

Beyond these transcriptional and signaling-level regulatory effects, increasing evidence suggests that the intrinsic cellular state of bone-resident cells—particularly the senescent status of bone marrow mesenchymal stem cells (BMSCs)—plays a critical role in determining osteogenic competence. Under diabetic conditions, senescent BMSCs exhibit enhanced secretion of senescence-associated secretory phenotype (SASP) factors, which amplify local inflammatory signaling and disrupt the osteogenic microenvironment, thereby indirectly impairing bone formation (115). Accordingly, therapeutic strategies aimed at delaying or modulating bone cell senescence have attracted growing attention. For instance, scutellarin (SCU) has been reported to markedly attenuate bone loss in DOP mouse models, an effect associated with reduced hyperglycemia-induced senescence in LepR^+^ bone marrow mesenchymal stem cells. Mechanistically, SCU facilitates sustained activation of the Nrf2-ARE antioxidant pathway through Ezh2-mediated repressive histone modification (H3K27me3) at the *Keap1* locus, thereby improving intracellular redox homeostasis and alleviating senescence-associated cellular stress. This epigenetically mediated antioxidant response may contribute to the preservation and partial restoration of BMSC osteogenic capacity (116).

#### 6.2.2 Suppression of excessive osteoclast activation and bone resorption

Chronic inflammation and the hyperglycemic microenvironment characteristic of diabetes markedly enhance osteoclast-mediated bone resorption. The interaction between RANKL and its receptor RANK represents the central initiating event in osteoclast differentiation and activation. Subsequent activation of downstream signaling pathways—including NF-κB and mitogen-activated protein kinases (MAPKs; p38, JNK, and ERK)—induces the expression of the master transcription factor NFATc1, thereby driving the transcription of osteoclast-specific genes.

A wide range of natural products effectively suppress osteoclastogenesis by targeting these signaling cascades. Specnuezhenide (SPN) inhibits RANKL-induced activation of NF-κB by preventing IκBα phosphorylation and p65 nuclear translocation, while simultaneously suppressing MAPK pathway phosphorylation, leading to downregulation of key osteoclastogenic transcription factors c-Fos and NFATc1 (117). Tubeimoside I similarly inhibits osteoclast differentiation through blockade of NF-κB signaling (118). Cryptotanshinone suppresses osteoclast precursor fusion and mature multinucleated osteoclast formation by directly binding to lymphocyte cytosolic protein 1 (LCP1) (119). Bruceine A (BA) interferes with RANKL-triggered inflammatory signaling in a Receptor for activated C kinase 1 (RACK1)-dependent manner by disrupting the interaction between RACK1 and c-Src proteins (120).

Regulation of the OPG/RANKL balance represents an upstream strategy to limit osteoclast activation at its source. Salidroside (121), *Marantodes pumilum* leaf aqueous extract (122), and *Gastrodia elata* aqueous extract (123) all increase the OPG/RANKL ratio while enhancing antioxidant capacity, thereby exerting protective effects against diabetic bone loss. Notably, inhibition of excessive osteoclast activity is a critical step in preventing osteoporosis progression. Biochanin A (BCA) significantly reduces osteoclast numbers and improves bone mass in a type 2 diabetic osteoporosis model by lowering ROS levels, suppressing excessive MAPK activation, and blocking NFATc1 expression (124).

Accordingly, simultaneously promoting osteogenesis while inhibiting osteoclastogenesis is regarded as a fundamental strategy for restoring bone metabolic homeostasis in DOP (as summarized in **Table 5**).

**Table 5:** Natural products regulating bone homeostasis in DOP.

Function/Natural product	Source/Components	Model	Mechanisms	References
Promoting osteogenesis				
Icariin	*Epimedium* spp.	*In vivo*: STZ-treated SD rats;	Upregulating RUNX2 and OPG/RANKL ratio; promoting BMSC osteogenic differentiation via GLI1	(98)
*Marantodes pumilum Leaf* aqueous extract	*Marantodes pumilum* (Merr.) Kiew	*In vivo*: STZ-treated SD rats;	Upregulating Wnt3a and β-catenin, downregulating GSK-3β; and improving bone structure.	(99)
Catalpol	*Rehmannia glutinosa* (Gaertn.) Libosch.	*In vivo*: HFD and STZ-treated ICR mice; *In vitro*: HG-treated MC3T3-E1 cells	Inhibiting KDM7A to relieve suppression of Wnt/β-catenin and promote osteogenesis.	(100, 101)
Liuwei Dihuang Pill (LWDH)	*Rehmannia glutinosa* (Gaertn.) DC., *Cornus officinalis* Siebold & Zucc., *Wolfiporia cocos* (F.A.Wolf) Ryvarden & Gilb., *Dioscorea oppositifolia* L., *Alisma orientale* (Sam.) Juz., *Paeonia suffruticosa* Andrews	*In vivo*: HFD and STZ-treated SD rats; *In vitro*: HG-treated MC3T3-E1 cells	Inhibiting KDM7A, activating Wnt/β-catenin, and promoting osteogenic differentiation.	(102–104)
Flavonoids from Rhizoma Drynariae (RDF)	*Drynaria fortunei* (Kunze ex Mett.) J. Sm.	*In vivo*: HG+HFD and STZ-treated SD rats; *In vitro*: HG-treated MC3T3-E1 cells	Upregulating BMP2, Smad4/5, and RUNX2 to activate the BMP2/Smad pathway.	(105)
Rehmanniae Radix Praeparata active components	*Rehmannia glutinosa* (Gaertn.) Libosch.	HFD and STZ-treated Wistar rats; HG-treated MC3T3-E1 cells	Activating BMP and IGF-1/PI3K/mTOR pathways to promote osteogenesis.	(106)
Baicalin	*Scutellaria baicalensis* Georgi	*In vivo*: HG+HFD and STZ-treated rats; *In vivo*: BMSC	Activating p38-MAPK to drive BMSCs toward osteogenic over adipogenic differentiation.	(107)
Anthocyanin-Rich Extract from Black Rice (AEBR)	*Oryza sativa* L. subsp. *japonica*	*In vivo*: STZ-treated rats	Dose-dependently inhibiting bone marrow adipogenesis and upregulating bone RUNX2 and OPG/RANKL ratio.	(108)
Anemarrhenae Rhizoma/Phellodendri Chinensis Cortex Herb Pair (AR/PCC) & Absorbed Components	*Anemarrhena asphodeloides* Bunge/*Phellodendron chinense* Schneid.	*In vivo*: STZ-induced zebrafish and GK rats; *In vitro*: HG-induced osteoblasts	Upregulating osteogenic genes (alp, runx2b, opg) and downregulating osteoclastic genes (acp5α, rankl, sost).	(109)
Quercetin	Various spp.	*In vivo*: STZ-treated rats	Improving bone density, microstructure, or osteogenesis and mitigating oxidative stress.	(110)
Cinnamaldehyde	*Cinnamomum cassia* (L.) J. Presl	*In vivo*: STZ-treated OVX mice	Improving bone density, microstructure, or osteogenesis targeting netrin-1/DCC/UNC5B pathway.	(111)
D-pinitol	*Leguminosae* spp.	*In vitro*: HG-treated osteoblasts	Improving bone density, microstructure, or osteogenesis via elevating tissue D-chiro-inositol levels.	(112)
Epimedium Polysaccharides	*Epimedium* spp.	*In vitro*: HG-treated osteoblasts	Improving bone density, microstructure, or osteogenesis by modulating Bax/Bcl2	(113, 114)
Scutellarin (SCU)	*Erigeron breviscapus* (Vant.) Hand.-Mazz.	*In vivo*: HFD and STZ-treated C57BL/6J mice	Reducing cellular senescence via Ezh2-mediated H3K27me3 on Keap1, leading to sustained Nrf2-ARE pathway activation	(116)
Inhibiting osteoclastogenesis				
Specnuezhenide (SPN)	*Ligustrum lucidum* W.T. Aiton	*In vivo*: HG+HFD and STZ-treated SD rats	Inhibiting RANKL-induced NF-κB activation and MAPK phosphorylation, and downregulating c-Fos and NFATc1	(117)
Tubeimoside I	*Bolbostemma paniculatum* (Maxim.) Franquet	*In vivo*: HG+HFD and STZ-treated SD rats; *In vitro*: RANKL-induced osteoclasts	Inhibiting NF-κB pathway to suppress osteoclastogenesis	(118)
Cryptotanshinone	*Salvia miltiorrhiza* Bunge	*In vivo*: HFD and STZ-treated C57BL/6 mice *In vitro*: HG-induced BMMs and RAW264.7 cell	Binding to LCP1, interfering with osteoclast precursor fusion	(119)
Bruceine A (BA)	*Brucea javanica* (L.) Merr.	*In vivo*: db/db mice; *In vitro*: RANKL-induced BMMs	Disrupting RACK1-c-Src interaction in a RACK1-dependent manner	(120)
Salidroside	*Rhodiola rosea* L.	*In vivo*: STZ-induced OVX SD rats	Upregulating OPG/RANKL ratio and enhancing antioxidant capacity	(121)
*Marantodes pumilum leaf* aqueous extract	*Marantodes pumilum* (Merr.) Kiew	*In vivo*: STZ-treated SD rats	Upregulating OPG/RANKL ratio and enhancing antioxidant capacity	(122)
*Gastrodia elata* water extract	*Gastrodia elata* Blume	*In vivo*: STZ-treated SD rats	Upregulating OPG/RANKL ratio and enhancing antioxidant capacity	(123)
Biochanin A (BCA)	*Cicer arietinum* L.	*In vivo*: db/db mice; *In vitro*: RANKL-induced BMMs	Reducing ROS, inhibiting excessive MAPK activation, blocking NFATc1, and decreasing osteoclast numbers	(124)

## 7 Natural products modulating multi-organ crosstalk and systemic regulation in DOP

**Figure 2:**
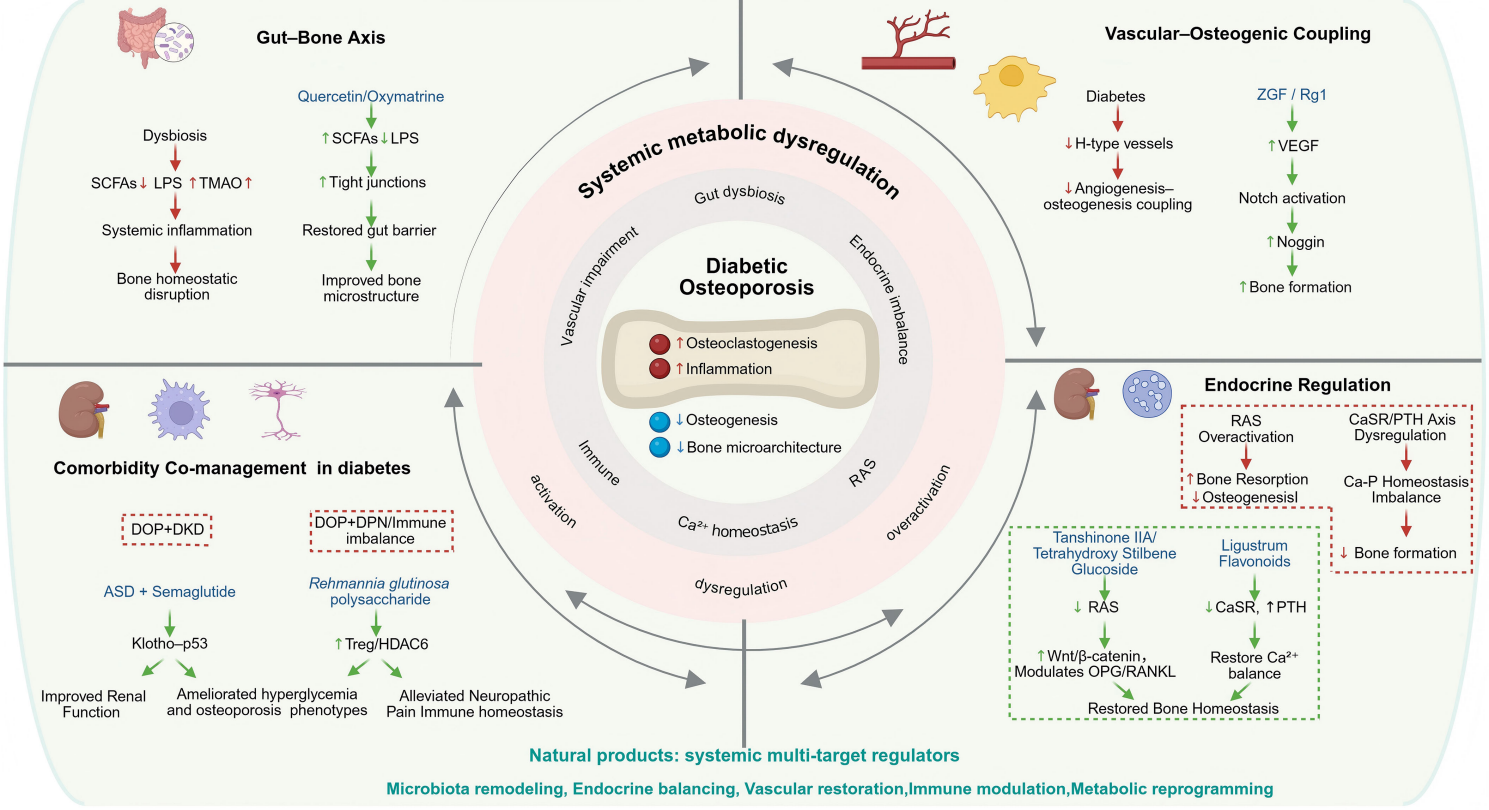
Systemic and multi-organ signaling crosstalk targeted by natural products in DOP. Red arrows indicate pathological activation; green arrows indicate protective regulation by natural products. Figure created with BioRender.com.

Beyond AGEs and ROS, multiple mediators—including inflammatory, endocrine, gut-derived, and epigenetic factors—are also implicated in DOP. The development of DOP is not confined to bone tissue itself but is embedded within a systemic context of metabolic dysregulation. The gut, vasculature, and other organs interact with the skeletal system through endocrine, immune, and metabolic networks, and dysfunction within these systems may collectively impair bone homeostasis (125, 126). Compared with single-target interventions, natural products, owing to their multi-component composition and systemic regulatory properties, may offer advantages in modulating inter-organ crosstalk and restoring overall metabolic balance, as summarized in **Table 6** and schematically illustrated in **Figure 2**. In addition, dietary intervention strategies such as intermittent fasting have been proposed to exert systemic effects through modulation of the gut–bone axis, providing new avenues for DOP prevention and treatment (127).

**Table 6:** Natural products modulating multi-organ crosstalk and systemic regulation in DOP.

Pathway/Natural product	Source/Components	Model	Mechanisms	References
Modulation of the gut-bone axis				
Quercetin	Various spp.	*In vivo*: HFD and STZ-induced C57BL/6J mice	Improving gut microbiota composition, enhancing SCFA metabolism, lowering lipids, and correlating with improved bone balance and microstructure	(130)
Oxymatrine (OMT)	*Sophora flavescens* Aiton	*In vivo*: HG+HFD and STZ-induced SD rats; *In vitro*: LPS-inducedosteoblasts	Altering gut microbiota and repairing intestinal barrier (upregulating Occludin, ZO-1); downregulating miR-539-5p in osteoblasts to activate the OPG/RANKL–Runx2 pathway	(131)
Restoration of vascular-osteogenic coupling				
Zhuanggu Fang (ZGF)	*Epimedium sagittatum* (Sieb. et Zucc.) Maxim., *Astragalus membranaceus* (Fisch.) Bge., *Dioscorea opposita* Kunb., *Eucommia ulmoides* Oliv., *Salvia miltiorrhiza* Bge., *Panax notoginseng* (Burk.) F.H. Chen	*In vivo*: GK rats	Increasing H-type vessel density, upregulating Osterix, and activating the VEGF/Notch1/Noggin pathway	(136)
Ginsenoside Rg1	*Panax ginseng* C.A. Meyer	*In vivo*: GK rats; *In vitro*: HG-induced osteoprogenitors and HUVECs	Promoting VEGF secretion and activating endothelial Notch signaling; stimulating H-type vessel formation and bone formation	(137)
Regulation of endocrine axes				
Tanshinone IIA	*Salvia miltiorrhiza* Bunge	*In vivo*: STZ-treated C57BL/6J mice; *In vitro*: Human embryonic kidney (HEK) 293 cells	Inhibiting renin activity and downregulating angiotensin II levels	(138)
Tetrahydroxy Stilbene Glucoside	*Polygonum multiflorum* Thunb.	*In vivo*: STZ-treated C57BL/6J mice; *In vitro*: MC3T3-E1	Inhibiting local RAS activity, promoting Wnt/β-catenin, and modulating the OPG/RANKL axis	(139)
Ligustrum Flavonoids	*Ligustrum lucidum* W.T. Aiton	*In vivo*: STZ-treated C57BL/6J mice	Elevating serum PTH and inhibiting calcium loss; potentially downregulating renal CaSR	(140)
Comorbidity co-management in diabetes				
Akebia Saponin D (ASD)	*Akebia quinata* (Thunb.) Decne.	*In vivo*: HG+HFD and STZ-induced SD rats	Synergistically improving diabetic nephropathy and osteoporosis, potentially via the Klotho–p53 axis	(141)
*Rehmannia glutinosa* polysaccharide (RGP)	*Rehmannia glutinosa* (Gaertn.) Libosch.	*In vivo*: STZ-treated mice	Ameliorating hyperglycemia, osteoporosis, and neuropathic pain, potentially via immune modulation (e.g., upregulating HDAC6 in Tregs)	(79)

### 7.1 Modulation of the gut-bone axis

Gut microbiota dysbiosis is a hallmark of DOP, characterized by reduced short-chain fatty acid (SCFA)-producing bacteria, expansion of pro-inflammatory taxa, and altered serum metabolite profiles, including decreased SCFA levels and elevated cholesterol (128, 129). These alterations affect bone through multiple mechanisms: SCFAs (e.g., acetate, butyrate) inhibit osteoclast differentiation via free fatty acid receptor 2 (FFAR2), whereas gut barrier dysfunction and dyslipidemi facilitate systemic inflammation through translocation of lipopolysaccharide (LPS), suppressing osteoblast function. Additionally, elevated trimethylamine N-oxide (TMAO) levels are associated with bone metabolic disorders and inflammatory status in diabetic nephropathy, indicating a critical role for the gut-kidney-bone axis in multi-organ crosstalk (125).

Several natural products have been shown to restore bone health by reshaping gut microbiota, enhancing gut barrier integrity, and regulating metabolite profiles. Combined administration of quercetin and dasatinib improves microbial composition in DOP mice, increasing SCFA-producing genera (e.g., *Lachnospiraceae*, *Bacteroides*), reducing pro-inflammatory bacteria (e.g., *Mucispirillum*), and normalizing amino acid and SCFA metabolism, collectively improving bone-fat balance and microstructure (130). Oxymatrine (OMT) reduces the abundance of LPS-releasing Gram-negative bacteria, repairs colonic mucosa, upregulates tight junction proteins (Occludin, ZO-1), and lowers circulating LPS. Mechanistically, OMT downregulates miR-539-5p in LPS-stimulated osteoblasts, relieving inhibition of osteoglycin (OGN), and subsequently activates the OPG/RANK–Runx2 pathway to promote osteogenesis (131). Together, these findings highlight the role of the gut–bone axis in balancing osteogenesis and osteoclastogenesis, and polysaccharides such as Yigu Decoction and Dendrobium officinale polysaccharides can exert similar osteoprotective effects through microbiota-mediated modulation of key metabolites and osteogenic pathways such as Wnt/β-catenin (132, 133).

### 7.2 Restoration of vascular-osteogenic coupling

Intraosseous vasculature, particularly H-type vessels (CD31^hiEmcn^hi), spatially associates with osteoprogenitor cells and underlies angiogenesis-osteogenesis coupling (134). Diabetes impairs this coupling, leading to insufficient skeletal blood supply and diminished osteogenic capacity (135). Natural products can restore vascular-osteogenic interactions and bone homeostasis.

Zhuang-Gu-Fang (ZGF) enhances H-type vessel density in the bone of GK rats, upregulates osteogenic transcription factor Osterix, and activates the Vascular Endothelial Growth Factor/Notch receptor 1/BMP antagonist Noggin(VEGF/Notch1/Noggin) signaling axis, improving bone microarchitecture and density (136). Ginsenoside Rg1 promotes VEGF secretion from osteoprogenitors, activates Notch signaling in endothelial cells, induces Noggin release, and forms a positive feedback loop enhancing vascular-osteogenic coupling, ultimately increasing bone formation in GK rats (137).

### 7.3 Regulation of endocrine axes

Endocrine dysregulation is another critical driver of DOP. Overactivation of the renin-angiotensin system (RAS) suppresses osteogenesis and promotes resorption. Tanshinone IIA inhibits renin activity, reduces angiotensin II levels in serum and bone tissue, and improves proximal tibial bone density, microstructure, and distal femoral trabecular area in diabetic mice (138). Tetrahydroxy stilbene glucoside from *Polygonum multiflorum* similarly alleviates DOP by inhibiting local RAS activity, promoting Wnt/β-catenin signaling, and regulating the OPG/RANKL axis, thus enhancing osteogenesis and inhibiting osteoclastogenesis (139).

Calcium-phosphorus homeostasis via the calcium-sensing receptor (CaSR) and PTH axis is another key regulatory pathway. The CaSR senses serum calcium levels and modulates PTH secretion, and its dysfunction contributes to diabetic bone loss. Ligustroflavone, a flavonoid from *Ligustrum lucidum*, elevates serum PTH, improves calcium balance by reducing urinary calcium excretion and bone calcium loss, and enhances bone density and trabecular microstructure. These effects may involve downregulation of excessive renal CaSR expression and restoration of PTH-mediated anabolic activity in bone (140).

### 7.4 Comorbidity co-management in diabetes

In the context of diabetes-associated multi-organ complications, combination therapies using natural products have demonstrated superior systemic regulatory potential^138]^. For instance, co-administration of Akebia saponin D (ASD) and the glucagon-like peptide-1 receptor agonist semaglutide markedly improves renal function, bone microarchitecture, and mechanical strength compared with monotherapy in a rat model exhibiting both diabetic kidney disease (DKD) and osteoporosis. Mechanistically, these effects may involve modulation of the Klotho-p53 signaling axis (141). Similarly, *Rehmannia glutinosa* Libosch polysaccharide (RGP) not only ameliorates hyperglycemia and osteoporosis phenotypes in streptozotocin-induced diabetic mice but also mitigates diabetic peripheral neuropathy (DPN)-related pain and improves nerve function. These protective effects appear partially mediated through regulation of systemic immune homeostasis, including upregulation of histone deacetylase 6 (HDAC6) expression in regulatory T cells (79). Collectively, these studies highlight the potential of natural product-based strategies for the synergistic prevention and management of multiple diabetic complications.

Taken together, these findings indicate that natural products can modulate DOP not only through bone-intrinsic mechanisms, but also by coordinating multi-organ crosstalk and systemic regulatory networks, as summarized in **Table 6** and illustrated in **Figure 2**.

## 8 Discussion

**Figure 3:**
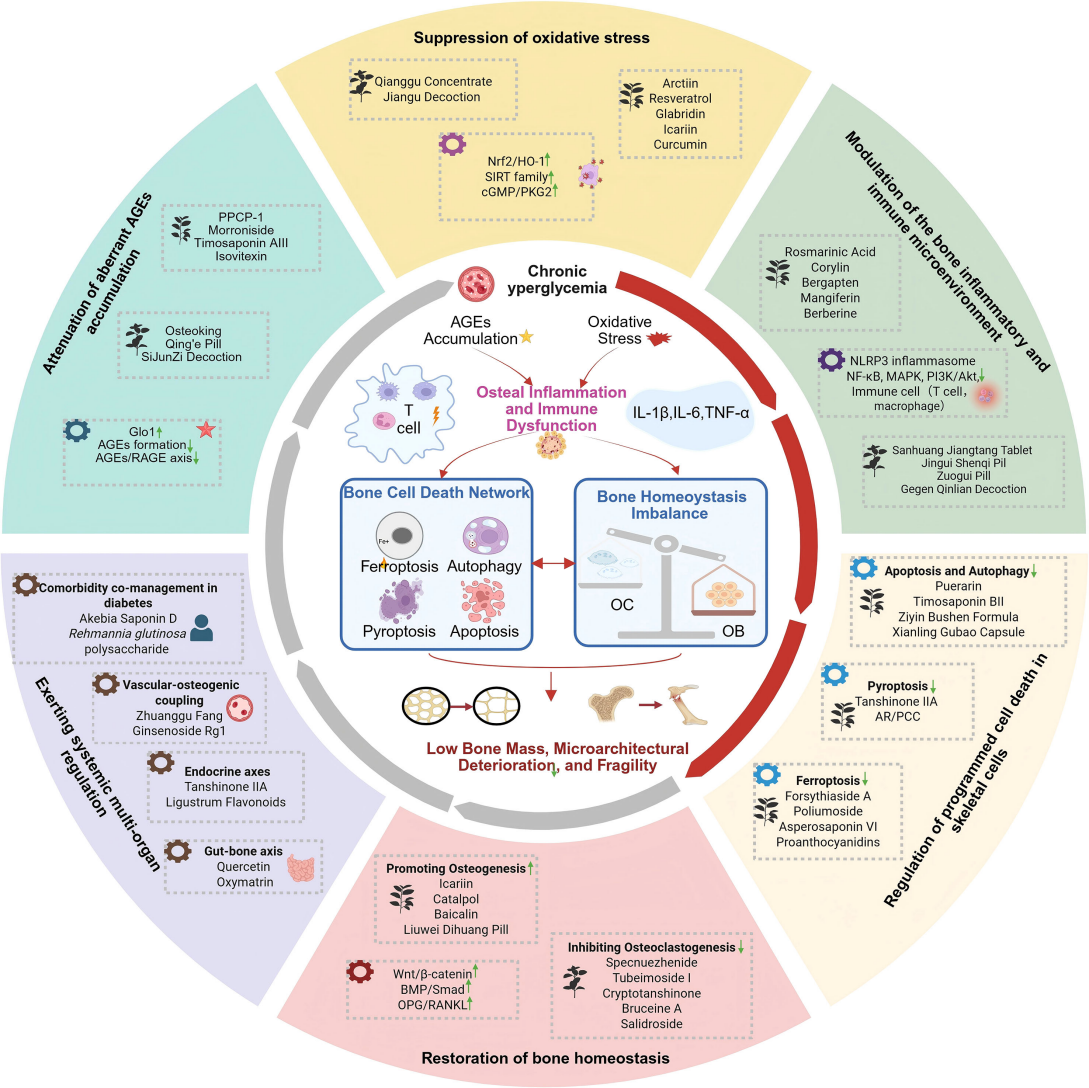
Integrated pathological network of DOP and multi-target modulation by natural products. Red arrows indicate pathological activation; green arrows indicate protective regulation. Figure created with BioRender.com.

As summarized in **Figure 3**, the development of DOP involves a complex interplay of hyperglycemia and AGEs accumulation, leading to oxidative stress, chronic inflammation, immune dysregulation, programmed cell death of bone cells, and disruption of the bone microenvironment. Recent studies suggest that natural products may exert effects on multiple pathological pathways simultaneously, mitigating AGEs toxicity, oxidative stress, and inflammatory imbalance, and potentially improving the function of bone-related cells under hyperglycemic conditions via epigenetic or senescence-related mechanisms. Some studies have also highlighted potential effects beyond bone tissue, such as modulation of gut microbiota and barrier function, promotion of H-type vessel formation within bone, or regulation of the renin–angiotensin system and CaSR–PTH endocrine axis, which may contribute to modulation of the bone microenvironment. DOP often coexists with chronic diabetic complications, including DPN and DKD, which may exacerbate bone metabolic imbalance via neural, endocrine, inflammatory, or metabolic pathways. Certain natural products may not only improve bone microstructure but also confer protective effects on nerve, kidney, or vascular function, providing experimental evidence for potential “neuro–bone” or “renal–bone” axes in multi-organ interventions. This underscores that DOP’s skeletal phenotype is closely linked to systemic metabolic status, and research should consider both bone formation/resorption and the influence of metabolic disorders and comorbidities, helping to explain the “bone density paradox” and inform multi-targeted intervention strategies.

Most mechanistic studies to date have relied on db/db, GK or STZ-induced rodent models and high-glucose-treated osteoblast/osteoclast cultures. Although these models are indispensable for mechanistic exploration, the evidence remains preclinical. Given the differences in metabolic background, disease progression, and comorbidity profiles between these models and clinical patients, the long-term efficacy and safety in humans remain to be determined. Clinical translation of natural products remains limited by low bioavailability and poor bone targeting. Emerging delivery strategies, such as ROS-responsive nanocarriers or bioactive nucleic acid vectors, can enhance bone-specific drug accumulation and release, and may synergistically suppress oxidative stress and cell death; however, their scalability, long-term safety, and applicability across disease models require further evaluation.

Notably, experience from metabolic and orthopedic disorders provides some reference points for the clinical development of natural products in DOP. For example, curcumin has entered clinical evaluation in osteoarthritis and metabolic diseases, and nanoformulations such as Theracurmin^®^ and Cavacurmin have addressed its limited bioavailability, allowing more consistent systemic exposure (142, 143). Berberine has also been tested in randomized controlled trials, where it improved glycemic control, lipid parameters, and insulin sensitivity with an acceptable safety profile (144, 145). In addition, multi-component formulations such as Osteoking (OK) have shown clinical benefits in fracture healing, including improvements in bone turnover markers and callus formation (146). Experimental studies suggest that OK activates Wnt/β-catenin signaling and regulates inflammatory responses—mechanisms that overlap, at least in part, with pathways implicated in DOP. While fracture repair and DOP arise from distinct pathological settings, these findings illustrate how standardized compound formulations can be advanced into clinical testing, rather than serving as direct evidence of efficacy in DOP itself.

As a narrative review, this article is based on non-systematic literature searching and may be subject to potential reporting bias. In DOP, the major obstacle is less about whether natural products have therapeutic potential, and more about the lack of a disease-oriented clinical translation strategy. A more pragmatic pathway may begin with mechanistic confirmation in well-characterized diabetic models, followed by small-scale clinical studies using oxidative stress indices, inflammatory mediators, and bone turnover markers as early readouts. Only thereafter should adequately powered randomized trials be designed to evaluate changes in bone density, microarchitecture, and fracture outcomes. At the same time, approaches such as multi-omics analyses and organoid-based models may help delineate how these agents act across different cell types and metabolic contexts and may assist in identifying patients more likely to benefit—for example, those characterized by elevated AGEs, distinct inflammatory signatures, or altered gut microbial metabolites. Such stepwise refinement would make the transition from preclinical observations to clinical testing in DOP more grounded and testable.

## 9 Conclusion

Accumulating evidence indicates that DOP is not driven by aberrations in a single signaling pathway but rather emerges from the sustained interplay among chronic metabolic dysregulation, persistent inflammatory stress, and progressive impairment of bone cell function. In this context, natural products and their bioactive constituents possess an inherent advantage by concurrently modulating multiple pathological nodes, a property that aligns closely with the multifactorial nature of diabetes-associated bone disorders. Nevertheless, the current body of evidence is still largely confined to *in vitro* systems and animal models, and critical questions regarding mechanism integration, effective dose windows, long-term safety, and translational relevance in human populations remain insufficiently addressed. Future investigations should therefore prioritize the integration of mechanistic networks, the development of advanced delivery and bone-targeting strategies, and the generation of high-quality, hypothesis-driven clinical evidence, in order to rigorously define the therapeutic value of natural products in the prevention and management of diabetes-related skeletal complications.
